# Neutrophil Extracellular Traps and Intestinal Diseases: A Narrative Review

**DOI:** 10.1155/grp/5908301

**Published:** 2026-03-03

**Authors:** Zhe Wang, Binni Yang, Zhenghong Li, Chen Gao

**Affiliations:** ^1^ Department of General Practice, The 940th Hospital of Joint Logistics Support Force of Chinese People’s Liberation Army, Lanzhou, China

**Keywords:** colorectal cancer, inflammatory bowel disease, intestinal microbiota, ischemia-reperfusion injury, neutrophil extracellular traps

## Abstract

Neutrophil extracellular traps (NETs) are a network‐like structure released by neutrophils into the extracellular space in response to various stimuli, with DNA as the main component, to which histones, granulins, cytoplasmic proteins, and other proteins are attached. Previous studies have shown that NETs are involved in the development of immune diseases, inflammation, tumors, etc. NETs are associated with the development of several intestinal diseases, including inflammatory bowel disease and intestinal ischemia–reperfusion injury. NETs may also interact with microorganisms to contribute to the development of inflammatory bowel disease and participate in the proliferation, metastasis, and thrombosis of colorectal cancer cells. This review comprehensively summarizes the latest research progress on NETs and intestinal diseases, providing a valuable reference for further investigations in this field.

## 1. Introduction

Neutrophils, the most prevalent type of granulocytes, accounting for 50%–70% of leukocytes, serve as the body’s frontline defense against foreign pathogens and are crucial in the innate immune response [1]. Immune effects are exerted in a variety of ways, including chemotaxis, phagocytosis, antimicrobial function, degranulation, and the formation of neutrophil extracellular traps (NETs) [2]. NETs are involved in different pathophysiological processes as a programmed cell‐specific death distinct from necrosis and apoptosis [3]. On one hand, they can act defensively by eliminating pathogens [4]; on the other hand, excessive NETs can lead to tissue or organ damage [5, 6]. An increasing number of studies have demonstrated that NETs are not only involved in infectious diseases, such as sepsis, but also participate in the pathophysiological processes of inflammation [7, 8], cardiovascular diseases [9], thrombosis [10], immune disorders [11], and organ damage [12]. This review focuses on recent research advances in NETs for inflammatory bowel disease (IBD), colorectal cancer (CRC), and intestinal ischemia–reperfusion injury.

## 2. Overview of NETs

### 2.1. Structure of NETs

In 1996, Takei et al. [13] stimulated neutrophils with the potent activator phorbol 12‐myristate 13‐acetate (PMA) and observed morphological changes distinct from typical apoptosis and necrosis. These changes included the fusion of the neutrophil’s lobulated nuclei, a reduction in the content and density of chromatin nuclei, and subsequent rupture of the nuclear membrane, while the organelles remained intact at this stage. Eight years later, Brinkmann et al. [14] further described this process in detail and named it NETs. NETs are formed by chromatin depolymerization, with DNA serving as the primary structure to which a variety of proteins are attached [15]. The proteins identified include citrullinated histone H3 (CitH3) [16], protease 3 (PR3) [17], lactoferrin [18], neutrophil elastase (NE) [19], gelatinase [20], cathepsin G (CG) [21], myeloperoxidase (MPO) [22], calprotectin [23], and over 30 others.

### 2.2. Formation of NETs

The process of NET formation is known as NETosis. It has been established that a wide range of chemical and biological factors, such as bacteria, fungi, viruses, parasites, lipopolysaccharide (LPS), interferons, and activated platelets, can stimulate neutrophils to produce NETs. The aforementioned autoimmune factors (e.g., autoantibodies) can induce NET formation, which explains the potential link between NETs and the immune disorder component of IBD [24]. Meanwhile, DNA methylation regulates NET formation, providing a molecular mechanism basis for targeting NETs in intestinal diseases [25].

**Figure 1:**
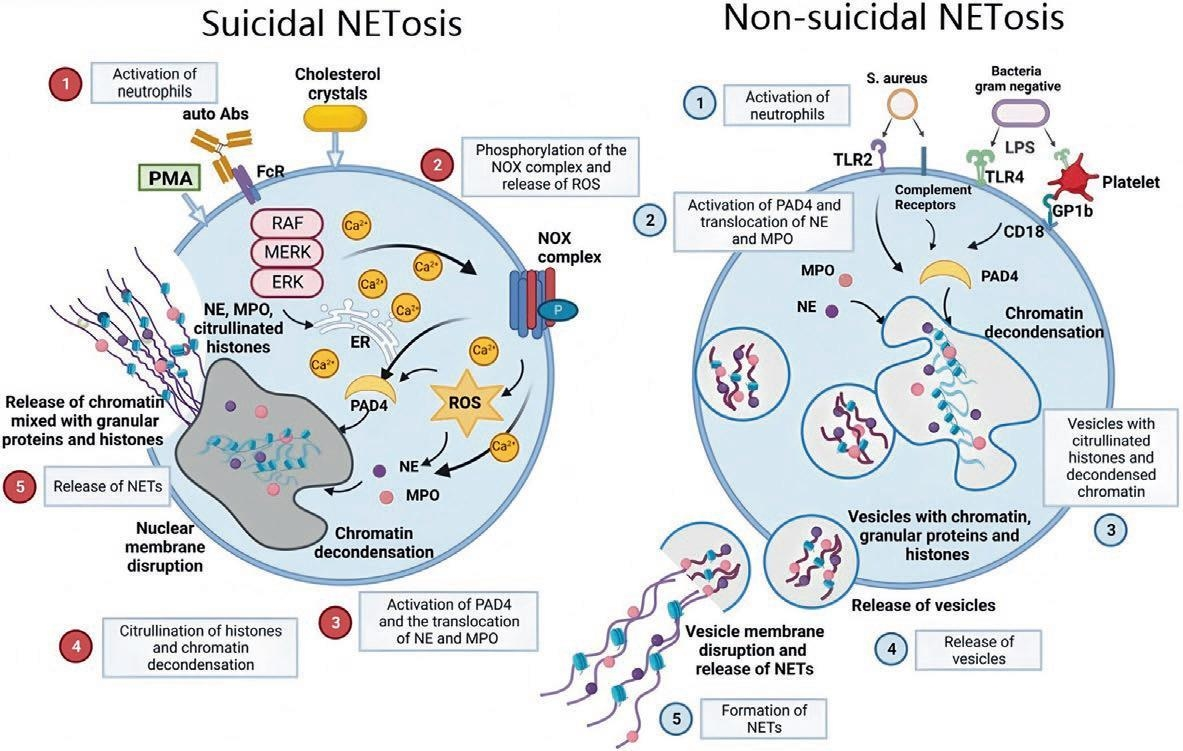
Sequential process of suicidal NETosis and nonsuicidal NETosis [7].

NETs are formed through different mechanisms depending on the stimuli, mainly divided into two types: suicidal and nonsuicidal (Figure 1). Suicidal and nonsuicidal NETosis constitute two sequential, mechanistically distinct pathways of NET formation. Figure 1 is adopted with modifications from [7], with permission from Springer Nature, Nature Medicine (2017).

#### 2.2.1. Suicidal NETosis

Neutrophils undergo death after NET formation. The process begins when neutrophils are stimulated by PMA, cholesterol crystals, etc., leading to the release of calcium from the endoplasmic reticulum into the cytoplasm. Through a series of signal transduction events, the Ras‐Raf‐MEK‐ERK and nicotinamide adenine dinucleotide phosphate (NADPH) oxidase pathways are activated, generating reactive oxygen species (ROS) [12, 26]. The activation of peptidyl‐arginine deiminase Type 4 (PAD4) promotes histone citrullination, resulting in chromatin depolymerization [27]. Simultaneously, activated MPO and NE migrate to the nucleus and act on histones, further contributing to chromatin depolymerization. Subsequently, the fibrous meshwork is expelled from the cell through the gasdermin D (GSDMD) membrane pore, ultimately leading to neutrophil death [28].

#### 2.2.2. Nonsuicidal NETosis

In this process, neutrophil death does not occur. Triggered by bacteria, Toll‐like receptors (TLRs), LPS, complement C3, etc. [12, 29], NETs are released through vesicles, and the neutrophil nuclear and cellular membranes remain intact [12, 30]. These neutrophils still retain the ability to chemotaxis, move, and phagocytose pathogens. However, the mechanism and physiological significance of nonsuicidal NETosis remain poorly understood and require further investigation.

## 3. NETs and IBD

### 3.1. NETs Promote IBD Progression

IBD encompasses a group of chronic nonspecific inflammatory bowel disorders with incompletely understood etiology and pathogenesis, including Crohn’s disease (CD) and ulcerative colitis (UC) [31]. The primary factors contributing to the onset and exacerbation of inflammation are inappropriate release of inflammatory mediators and impaired intestinal barrier function [32]. Proteomic analysis of UC biopsies showed elevated levels of 11 proteins that are enriched in NETs (e.g., MPO, NE, and citrullinated histones). However, since these components can also derive from intact or apoptotic neutrophils, this finding alone does not confirm the extracellular release of NETs. Boeltz et al. [12] systematically sort out the mechanism controversies and research status of NET formation and details of the differences in molecular pathways between suicidal NETosis and nonsuicidal NETosis and discuss the influence of different stimulating factors (e.g., bacteria, LPS, and PMA) on the NET formation pathway. Subsequent immunohistochemical and microscopy studies have provided additional support for genuine NET formation at the mucosal surface [27, 33]. Using conventional immunohistochemistry, Schroder et al. [34] observed higher mucosal staining for NE, MPO, and CitH3 in CD specimens with advanced histopathological scores. Because these proteins can reside within intact neutrophils or be released by degranulation—and because IHC cannot resolve their extracellular localization—this pattern does not, by itself, confirm the presence of NETs. A definitive demonstration requires the colocalization of NET‐core proteins with extracellular DNA, as observed by confocal or electron microscopy. Moreover, formation and expression of NETs were positively correlated with the severity of CD histopathology. Zhang et al. [35] reported elevated CitH3 and PAD4 levels, along with increased extracellular DNA, in the colons of mice with TNBS‐induced colitis. However, the study relied solely on Western blot and ELISA for CitH3 and PAD4 quantification; no immunofluorescence colocalization of extracellular DNA with NE or MPO was performed. Consequently, these data indicate heightened PAD4 activity and citrullination but do not constitute direct evidence of NET formation. The PAD4 inhibitor blocked NET formation, which in turn reduced the expression of proinflammatory factors, such as tumor necrosis factor‐alpha (TNF‐*α*); interleukin‐1*β*, 6, and 17A (IL‐1*β*, IL‐6, and IL‐17A); and interferon‐gamma (IFN‐*γ*). In contrast, anti‐inflammatory factors such as interleukin‐10 (IL‐10) were increased compared to the control. This suggests that the formation of NETs may promote the release of inflammatory mediators, and inhibiting PAD4 could potentially serve as a therapeutic target for IBD by blocking NET formation and reducing inflammation. Circulating extracellular DNA (ecDNA) is associated with poor prognosis in many diseases, and ecDNA released from neutrophils during infection or inflammation exists in the form of NETs [36]. In dextran sulfate sodium (DSS)–induced colitis mice, plasma ecDNA concentration was directly proportional to the severity of IBD, accompanied by an elevated endoscopic damage score and increased NET percentage [37]. Thus, NETs and their key components, including NE, MPO, and CitH3, are overexpressed in IBD and contribute to the release of proinflammatory factors, with their levels correlating with disease severity.

Impaired intestinal barrier function stems primarily from the disruption of intercellular tight and adherens junctions, together with epithelial cell death, thereby increasing paracellular and overall mucosal permeability, facilitating the entry of bacteria and toxins into the peritoneal cavity and triggering an immune‐inflammatory response, which is a crucial mechanism for aggravating intestinal inflammation in IBD, in which NETs are involved [38]. Jorch and Kubes [7] first proposed the role of NETs in noninfectious diseases, including intestinal inflammation, which laid the theoretical foundation for our focus on intestinal diseases (rather than only infectious diseases). Honda and Kubes [8] focused on the role of NETs in the liver and gastrointestinal system. The article detailed the research progress of NETs in digestive system diseases such as IBD and nonalcoholic fatty liver disease. It pointed out that in the intestinal mucosa of IBD patients, NETs can exacerbate intestinal inflammation by disrupting the tight junctions of the intestinal epithelium and promoting the release of inflammatory factors. Lin et al. [39] found that NET formation and expression were enhanced in mice with colitis induced by DSS and TNBS, resulting in damage to intestinal permeability, shifted intestinal flora, and exacerbated inflammation. Euler and Hoffmann [5] elaborated on the “double‐edged sword” role of NETs in inflammation—they not only participate in pathogen clearance as part of the innate immune defense but also cause tissue or organ damage due to their excessive formation. Additionally, NETs induced epithelial cell apoptosis and disrupted the tight junctions and adherent junctions between cells [40]. However, treatment with Deoxyribonuclease I (DNase I) restored the integrity of the intestinal mucosal barrier and alleviated intestinal inflammation in DSS mice compared to the control group. Therefore, NETs play a detrimental role in the dysfunction of the intestinal epithelial barrier, exacerbating inflammation in acute colitis. Li et al. [41] have demonstrated that NETs play a central role in the development of IBD, mediating macrophage activation and the release of proinflammatory cytokines, including TNF‐*α* and IL‐6, which ultimately leads to intestinal injury. Thus, NETs promote IBD progression by impairing the function of the intestinal mucosal barrier, and inhibiting NET formation may be a potential therapeutic strategy for IBD.

### 3.2. Interaction Between NETs and Intestinal Microbiota

The intestinal microbiota, comprising bacteria, fungi, viruses, and other microorganisms, is a vital component in maintaining host health and plays a crucial role in shaping the innate and adaptive immune systems in the mucosa [42, 43]. Dysregulation of the microbiota can trigger abnormal host immune responses, characterized by the intense recruitment and activation of neutrophils in the lamina propria of the crypts and the intestinal epithelium, resulting in tissue damage. It has been demonstrated that microorganisms and their products can influence the phenotypic and functional differentiation of neutrophils as well as the release and inhibition of NETs [44]. Vong et al. [45] found that *adherent-invasive Escherichia coli* exacerbated intestinal dysbiosis after antibiotic use while promoting the generation of NETs and ROS. Thus, pathogenic bacteria can enhance NET formation and local inflammatory responses in the intestine. Li et al. [46] discovered that butyrate produced by probiotics inhibited neutrophil migration and NET formation in CD and UC patients. In DSS‐induced colitis mice, oral butyrate was shown to suppress neutrophil‐related immune responses and formation and to improve mucosal inflammation. Therefore, probiotics and their metabolites can inhibit NET formation and may hold promise in the treatment of IBD [47]. Based on these studies, it is evident that pathogenic bacteria can promote the inflammatory response in IBD by inducing NET formation, while probiotics and their products can modulate the immune response of the intestinal mucosa and repair damaged mucosa by suppressing NET formation. Therefore, probiotics may be considered as a means to inhibit NET generation, thereby assisting the treatment of IBD.

**Figure 2:**
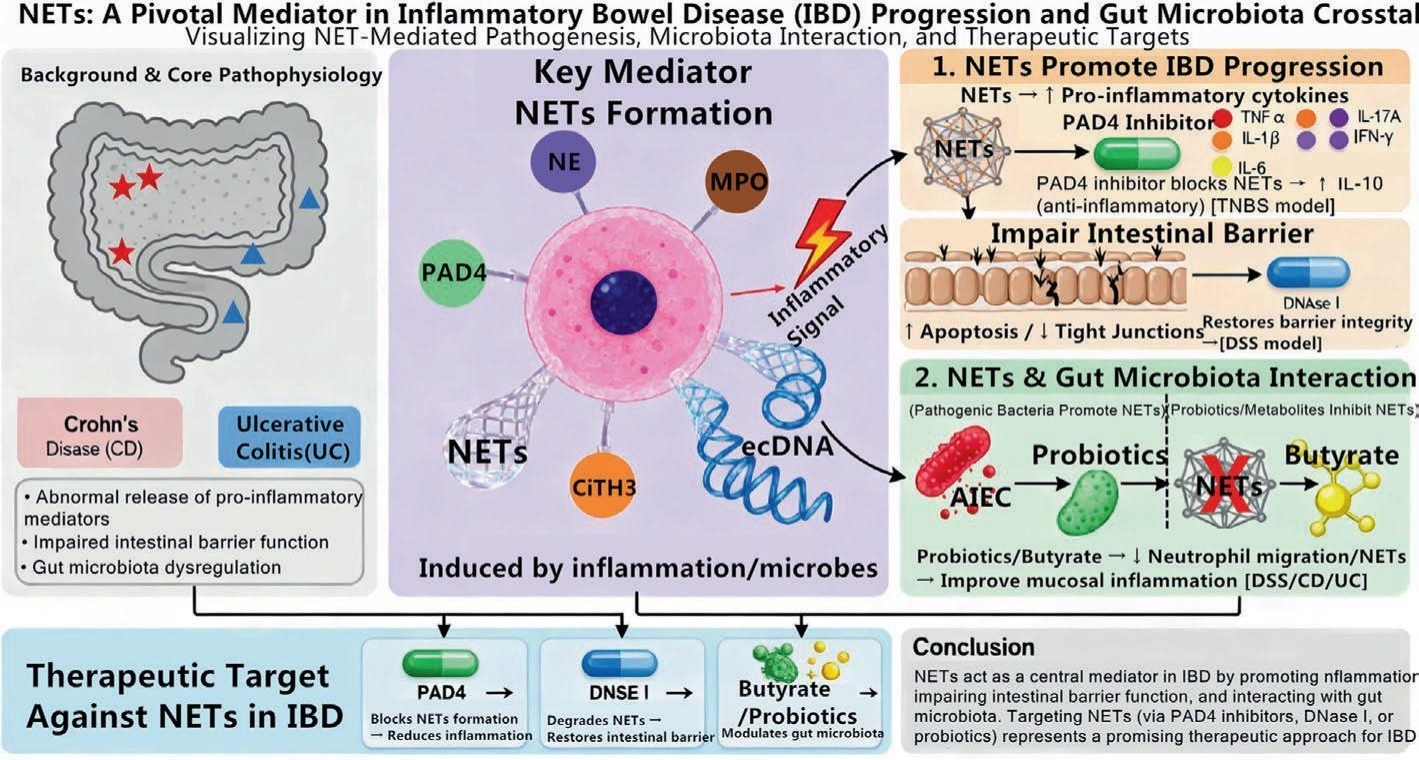
Mechanism overview: figure of NETs and IBD. Note: The figure is an original figure created by the authors of the manuscript.

The key content of Section 3 is visually presented through a mechanism overview figure (Figure 2).

## 4. NETs and CRC

### 4.1. NETs Promote CRC Proliferation

According to 2020 statistics, CRC has emerged as the leading cause of incidence and mortality in digestive system cancers, posing a significant threat to human health. In China, the incidence and mortality rates of CRC are relatively high globally [48, 49]. With the accelerating aging population and changes in lifestyle and dietary habits, the incidence of CRC is expected to continue rising, becoming a major public health concern. Yazdani et al. [50] found that NETs promoted cancer cell proliferation by enhancing the mitochondrial function of tumor cells and providing additional energy for tumor growth. The mechanism involves activating the release of NE by NETs, which activates Toll‐like receptor 4 (TLR4) on cancer cells, leading to the upregulation of peroxisome proliferator‐activated receptor *γ* coactivator 1‐alpha (PGC1*α*) and increased mitochondrial biosynthesis. In summary, NETs can stimulate tumor cell proliferation by enhancing the metabolic processes of cancer cells. NETs can also interact with the tumor microenvironment to prevent tumor cell death and promote cell proliferation, mainly through the formation of an immunosuppressive microenvironment and immune escape. Recent studies have shown [51] that NETs can encapsulate tumor cells, rendering T cells and NK cells in the immune response unable to recognize and kill the tumor cells. Thus, NETs can promote the proliferation of CRC cells by enhancing mitochondrial energy production and facilitating immune escape, suggesting that NETs may be a potential therapeutic target for rectal cancer [52].

### 4.2. NETs Promote CRC Metastasis

Tumor metastasis, the process by which cancer cells spread from the primary site to other tissues and organs and continue to grow, is a major obstacle to successful cancer treatment and a critical challenge that needs to be urgently addressed. CRC commonly metastasizes to the liver and is a leading cause of patient mortality. Analysis of clinical data from CRC patients has revealed a significant association between NET formation and metastasis and survival rates, although the underlying mechanism remains incompletely understood. Studies in mouse models have demonstrated that NETs promote cancer metastasis [53]. Observational studies have also indicated a close correlation between NETs and disease progression in CRC patients [54]. Pastor et al. [55] showed that the markers of NETs, including NE, MPO, and ecDNA, were significantly higher in patients with CRC metastases compared to healthy controls, suggesting a potential link between NETs and tumor metastasis. Additionally, it was found that Kirsten rat sarcoma viral oncogene homolog (KRAS) mutations induced interleukin‐8 (IL‐8) expression, which in turn increased NET formation and subsequently promoted CRC liver metastasis [56]. Rayes et al. [57] confirmed through CRC mouse model experiments that NETs can capture circulating tumor cells, facilitating the adhesion of CRC cells in lung and liver tissues and promoting tumor metastasis. Blocking NET formation through various approaches significantly inhibited tumor metastasis. In CRC mice injected with LPS, it was demonstrated that cancer cells can promote NET formation through the Toll‐like receptor 9 (TLR9) and mitogen‐activated protein kinases (MAPKs) signaling pathway. The metastatic ability of CRC can be attenuated by using DNase I [58]. Xia et al. [59] established a mouse model of CRC liver metastasis using an adeno‐associated virus (AAV) gene therapy vector that specifically expresses DNase I in the liver, demonstrating that AAV‐mediated DNase I liver gene transfer can inhibit liver metastasis in model mice. Therefore, AAV‐mediated DNase I liver gene transfer represents a safe and effective approach to inhibit metastasis, offering a novel therapeutic strategy for CRC. In conclusion, NETs can promote CRC metastasis through multiple mechanisms, and inhibiting NET formation using DNase I and other agents can reduce CRC metastasis and associated morbidity and mortality, providing new insights for CRC treatment.

### 4.3. NETs Promote Thrombosis in CRC

To date, metastasis and cancer‐related thrombosis remain the leading causes of death in CRC patients [25]. Cancer‐associated thrombosis is associated with a poor prognosis, and patients with CRC have an increased risk of venous thrombosis, although the exact mechanism remains unclear. NETs were first observed in deep vein thrombosis (DVT) in 2010, and it was established that NETs are involved in the development of DVT [60, 61]. Bonaventura et al. [9] focus on the association between NETs and cardiovascular diseases. Through clinical case analysis and in vitro cell experiments, MPO in NETs can activate the coagulation system and promote thrombosis. Elevated NETs levels can promote cancer‐related thrombosis [62]. One of the main mechanisms by which NETs promote thrombosis is by forming scaffolds that adhere to platelets, erythrocytes, and platelet adhesion molecules. Many of its components, such as NE, CG, and histones, can also promote platelet activation, aggregation, and blood coagulation. NETs can expose platelets to phosphatidylserine (PS), leading to a significant increase in procoagulant activity. They can also induce the conversion of endothelial cells to a procoagulant phenotype as well [9, 63]. Zhang et al. [64] demonstrated that NETs promote procoagulant activity in CRC patients, which in turn shortens coagulation time and increases thrombin–antithrombin complexes and fibrinofibrils. Therefore, NETs and their components can promote CRC‐related thrombosis, providing potential therapeutic targets to counteract the thrombotic consequences of CRC.

**Figure 3:**
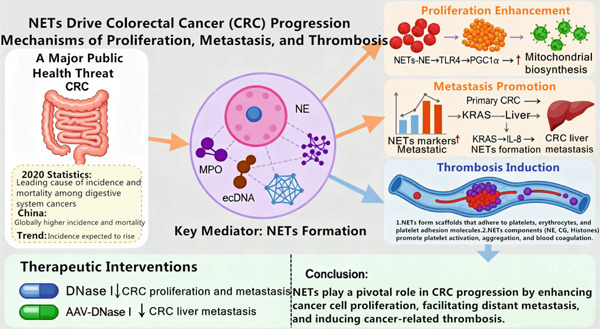
Mechanism overview: NETs and CRC. Note: The figure is an original figure created by the authors of the manuscript.

The key content of Section 4 is visually presented through a mechanism overview figure (Figure 3).

## 5. NETs and Intestinal Ischemia–Reperfusion Injury

Intestinal ischemia–reperfusion injury occurs when blood flow is restored after a period of intestinal ischemia, exacerbating intestinal damage and potentially leading to irreversible injury. It is commonly observed after acute mesenteric ischemia, shock, burns, and surgery. This condition can result in massive release of inflammatory mediators, displacement of the intestinal microbiota, endotoxin translocation, and damage to multiple organs, such as the heart, liver, and kidney, leading to the development of systemic inflammatory response syndrome, multiple organ dysfunction syndrome, and even death [65]. Czaikoski et al. [6] confirmed that NETs induce organ damage in both experimental sepsis and clinical sepsis. Kiwit et al. [66] demonstrated that in a rat model of ischemia–reperfusion injury, treatment with DNase I reduced intestinal neutrophil infiltration, as well as histone and MPO levels, and improved intestinal injury in rats. Therefore, NETs may contribute to intestinal ischemia–reperfusion injury. Ascher et al. [67] found that NET formation and expression in the mesenteric veins were enhanced after ischemia–reperfusion injury in a mouse model. Hayase et al. [68] reported increased histone and NET formation and NETs in the liver and intestine of mice with intestinal ischemia–reperfusion injury, accompanied by exacerbated hepatic injury. And recombinant thrombomodulin proteins could reduce liver injury by inhibiting histone and NETs. Therefore, NETs in intestinal ischemia–reperfusion injury not only aggravate local damage but also contribute to damage in other tissues and organs. In conclusion, NETs play a significant role in exacerbating local and systemic damage in intestinal ischemia–reperfusion injury and are involved in the progression of the disease.

**Figure 4:**
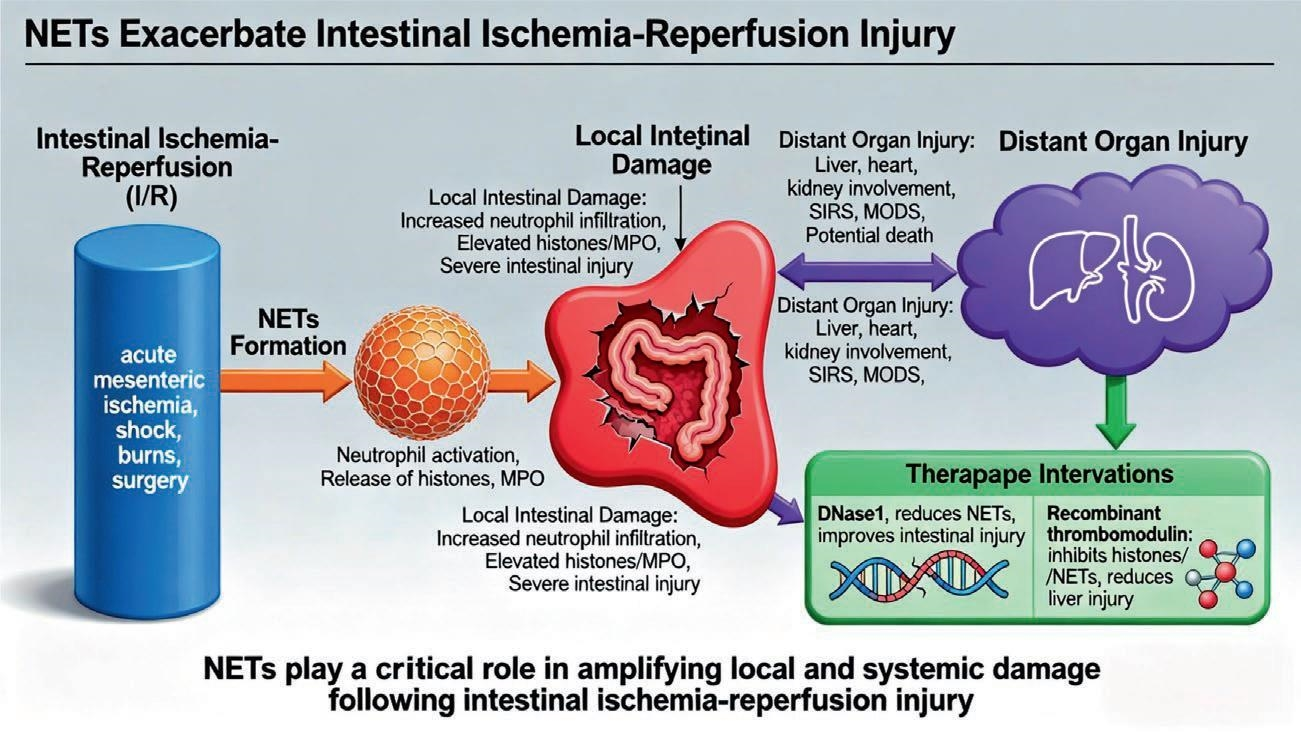
Mechanism overview: NETs and intestinal ischemia–reperfusion injury. Note: The figure is an original figure created by the authors of the manuscript.

The key content of Section 5 is visually presented through a mechanism overview figure (Figure 4).

## 6. Conclusions

NETs have emerged as a research focus in recent years, and their role in intestinal diseases cannot be overlooked. Studies have demonstrated that NETs are involved in the progression of IBD and interact with microorganisms in the pathophysiological process. NETs promote the proliferation and metastasis of CRC cells, as well as the formation of cancer‐related thrombosis. They are highly expressed in intestinal ischemia–reperfusion injury and contribute to the spread of the disease to other organs. Both in vitro and in vivo studies have shown that the use of PAD4 inhibitors and DNase I can inhibit NET formation, alleviating the disease and potentially serving as therapeutic targets. However, the precise mechanisms underlying the involvement of NETs in these diseases remain to be fully elucidated and require further in‐depth investigation.

## Author Contributions

Formal analysis: Binni Yang. Funding acquisition: Chen Gao. Writing—original draft: Zhe Wang. Writing—review and editing: Zhenghong Li and Chen Gao. Zhe Wang and Binni Yang contributed equally to this work.

## Funding

The study was funded by the Natural Science of Foundation of Gansu Province, China (23JRRA1668) and the Foundation of 940th Hospital Research Project, Lanzhou, Gansu Province, China (2023YXKY037).

## Disclosure

All authors have read and agreed to the published version of the manuscript.

## Conflicts of Interest

The authors declare no conflicts of interest.

## Data Availability

Data sharing is not applicable to this article as no datasets were generated or analyzed during the current study.
